# Organization of Cellular Receptors into a Nanoscale Junction during HIV-1 Adhesion

**DOI:** 10.1371/journal.pcbi.1000855

**Published:** 2010-07-15

**Authors:** Terrence M. Dobrowsky, Brian R. Daniels, Robert F. Siliciano, Sean X. Sun, Denis Wirtz

**Affiliations:** 1 Department of Chemical and Biomolecular Engineering, The Johns Hopkins University, Baltimore, Maryland, United States of America; 2 Howard Hughes Medical Institute and Department of Medicine, The Johns Hopkins School of Medicine, Baltimore, Maryland, United States of America; 3 Department of Mechanical Engineering, The Johns Hopkins University, Baltimore, Maryland, United States of America; 4 Johns Hopkins Physical Sciences in Oncology Center, The Johns Hopkins University, Baltimore, Maryland, United States of America; Emory University, United States of America

## Abstract

The fusion of the human immunodeficiency virus type 1 (HIV-1) with its host cell is the target for new antiretroviral therapies. Viral particles interact with the flexible plasma membrane via viral surface protein gp120 which binds its primary cellular receptor CD4 and subsequently the coreceptor CCR5. However, whether and how these receptors become organized at the adhesive junction between cell and virion are unknown. Here, stochastic modeling predicts that, regarding binding to gp120, cellular receptors CD4 and CCR5 form an organized, ring-like, nanoscale structure beneath the virion, which locally deforms the plasma membrane. This organized adhesive junction between cell and virion, which we name the viral junction, is reminiscent of the well-characterized immunological synapse, albeit at much smaller length scales. The formation of an organized viral junction under multiple physiopathologically relevant conditions may represent a novel intermediate step in productive infection.

## Author Summary

The entry of human immunodeficiency virus (HIV) into cells is the target for new therapies preventing HIV infection. While intermediate steps of viral entry have been characterized, the progression between these steps and how they result in productive infection are not well understood. By using stochastic modeling, we examine the initial interaction of a single viral particle with a flexible plasma membrane populated with viral receptors. The model predicts the formation of an organized receptor ultrastructure beneath the viral particle, which we name viral junction and which may contribute to productive viral infection. The organization of the viral junction depends on receptor density, CD4 bond stability, membrane mechanical flexibility, as well as viral protein organization and density.

## Introduction

Strategies for antiretroviral therapy have recently focused on inhibiting human immunodeficiency virus (HIV) adhesion, fusion, and entry. The biochemical properties of the dynamic binding interactions between host cell and viral receptors have been well characterized [1], [2]. However, how these interactions may work together for viral adhesion to progress toward an effective fusion event is not well understood. In particular, whether a single viral particle has the ability to spontaneously organize receptors at the cellular plasma membrane is unknown. Viral adhesion occurs on length and time scales that are difficult to monitor in real time because of the limited spatial and temporal resolution of current light and electron microscopes and the small size of virions (100nm in diameter, with entry into the cell taking place after only a few minutes [3], [4]). Here we use stochastic modeling to test the fundamental hypothesis that the cell-virus interfacial area forms an organized ultrastructure during viral adhesion, similar to the well-characterized immunological synapse [5] but at much smaller length scales (i.e. 0.1µm versus 10µm, respectively [3], [6]).

Virus-cell adhesion is primarily governed by bimolecular bonds formed between the viral surface protein gp120, and its cellular receptor CD4 [7]. gp120 molecules are arranged on the viral surface in trimers [8], [9]. For type-1 HIV (HIV-1), productive infection is also dependent on the subsequent binding of a cellular co-receptor, most commonly CCR5 or CXCR4 [10]. gp120-coreceptor binding induces a dynamic refolding of viral surface proteins which provides the driving force for fusion of the viral and cellular membranes [11], [12]. The formation of lipid microdomains on length scales similar to the viral diameter have been shown to result in protein colocalization [13]. Here we hypothesize that viral protein adhesion to cellular receptors, coupled with plasma membrane rigidity can produce highly organized protein structures at the cell surface on the same length scales as the virion and lipid rafts [13].

**Figure 1:**
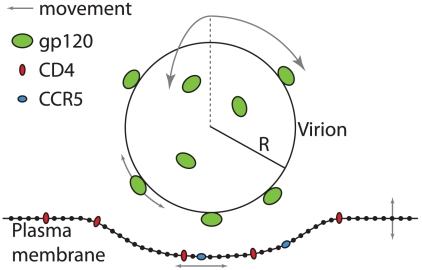
System detailing interaction of a viral particle with a flexible plasma membrane. The plasma membrane was modeled using Cartesian coordinates to evaluate the positions of discrete points. The plasma membrane was also populated with primary, CD4 (red), and secondary, CCR5 (blue), cellular receptors for HIV-1. The viral particle was modeled as a rigid sphere with radius, 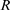 = 50nm, and was populated by gp120 trimers (green) capable of binding up to 3 CD4 and 3 CCR5 molecules. All entities were prohibited from diffusing through one another. The viral particle was allowed to move in 3 dimensions as well as rotate about its center, proteins were allowed to move in 2 dimensions along their respective surfaces and discrete plasma membrane points were allowed to move in 1 dimension either up or down. Movement directions within the plane here are shown with grey arrows.

We used stochastic modeling based on recent single-molecule force spectroscopy measurements [14] to assess the spatial and temporal organization of cellular receptor CD4 and co-receptor CCR5 at the plasma membrane, as they dynamically interacted with gp120 on the viral surface (Fig. 1). We will discuss how the energy of bond formation between gp120 on the virion and receptors on the cell surface acts as an organizing force amidst disordering thermal energy. Specifically, thermal energy drives stochastic movement of the laterally diffusing receptors on the plasma membrane, the formation and destruction of bonds between viral proteins and cellular receptors, and the deformation of the plasma membrane. The conditions that we explore here are designed to determine how each type of stochastic movement contributes to the organization of cellular receptors at the plasma membrane. In addition, we study whether viral particles with gp120 trimers that are capable of diffusing on the viral surface result in distinct receptor organization between the virus and the cell.

The combined system of virion, plasma membrane, viral receptors and cellular receptors studied here, is modeled as a succession of discrete states. The transition to new states is assumed to be a Markov process [15] and computed using the local-steady state approximation to the Fokker-Planck equation [16]. The organizations of virus-cell bonds discussed here were produced using the 3-D location of receptors on the plasma membrane actively bound to gp120 molecules relative to the center of the virion itself.

## Methods

We performed simulations considering the dynamic progress of the junction between the virion and the cell surface to be a stochastic, Markov process [15]. The system itself consists of a rigid sphere (the 100nm-diameter virion) interacting with a deformable surface (the plasma membrane, 200×200nm in dimension). The virion is populated with gp120 trimers, which can bind receptors (CD4 and CCR5) located on the plasma membrane (Fig. 1). All entities within the system including the plasma membrane, the configurations of viral proteins, the position of the virion itself, the dynamics of the bonds between the virus and the cell, as well as the positions of the cellular receptors (which are either bound or unbound to gp120), are specified by discrete states in Markov dynamics. The probabilities of generating a particular sequence of states, or a trajectory, are governed by the transition rates between these states. The transition rates between states that involve changes in the physical configuration of the system (i.e. the movement of the virion, the positions of the proteins and the plasma membrane from their current position to each possible new position) were calculated according to the local-steady state approximation of the Fokker-Planck equation [16],
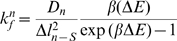
Here, 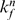 is the forward association rate for the 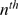 possible state adjacent to the current state (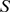), 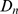 is the diffusion coefficient of each physical entity of the system (membrane proteins, viral proteins, etc.) whose position has changed between the current and future state, 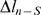 is the change in position of that physical parameter, 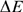 is the difference in system energy which results from the movement of that physical parameter between states and 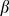 is 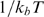 where 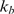 is Boltzmann constant and 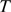 is the absolute temperature. This approximation calculates the rate of transition between the current state and subsequent adjacent state using the total change in energy that accompanies the progression from one discrete state to another. Specifically, a favorable change in energy (e.g. the relaxation of a bond between viral proteins and cellular receptors) results in an increased transition rate and an unfavorable change in energy (e.g. the deformation of the plasma membrane) results in a decreased transition rate from one state to the next.

Each possible state differs from the current state by the discrete position of any physically real object within the system (e.g. the x, y, z position of the virion, the position of a CD4 protein or a CCR5 protein, a discrete point along the plasma membrane, etc.) or by the creation or destruction of a bond between a receptor and gp120. For example, a single CD4 protein with no neighboring proteins on the plasma membrane would offer four possible new states based on its physical location because of the Cartesian coordinates used to define the flexible plasma membrane. In this example, 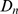 is an experimentally determined diffusion coefficient for CD4 on a cellular membrane, 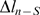 is the three-dimensional distance between the current position of CD4 and that of each available discrete position within the plasma membrane. For an unbound CD4 protein, a new state dictating the movement of the protein would use 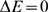. However, an unbound CD4 protein may also offer additional states which do not correspond to the physical movement of the CD4 if it is capable of binding an unbound gp120 protein. Those additional states will vary from the current state by the creation of a previously nonexistent bond; the forward rates toward these states are discussed below. However, should the CD4 protein in question already be participating in an existing bond, the forward rate corresponding to the movement of the bound CD4 protein would have a nonzero 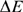 between states. This nonzero 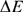 is the change in energy of the existing bond and determines the probability that this CD4 protein moves in an energetically favorable (relaxing the existing bond) or an energetically unfavorable (applying tension or compression to the bond) manner. For the case of receptors located on the plasma membrane, the forward rate constant is calculated using a 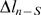 determined by the distance between discrete points along the plasma membrane, which vary during the simulation according to the membrane deformation. For system parameters such as the virion position, 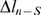 is a fixed step size which dictates that the forward rates will vary only according to the change in energy of the existing bonds. Similarly, the transition rates involving a change in z-position of discrete plasma membrane points are calculated using a fixed 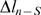. Here the z-position corresponds to the height of each plasma membrane point in the z-axis while the plasma membrane itself is oriented in the x-y plane. A fixed 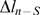, dictates that the transition rates of plasma membrane points will vary only according to the local membrane free energy, as discussed below, and if it be the location of a bound cellular receptor, the change in energy of that particular bond.

For the systems examined here, the elapsed time between states and the distance over which physical objects move are of such a small order of magnitude that two assumptions can be made. First, the local energy landscape is approximated to be linear. Second, the probability density is assumed to be at a local steady state. Therefore, at small length scales, the system of the virion and plasma membrane is well described by the high-friction limit of the Fokker-Plank equation.

The on and off rates (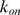 and 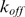) for CD4 and CCR5 bond formation with gp120 were calculated using experimentally measured rates [14]. Initial 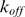 values, 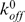, were computed using a model described by Hummer and Szabo [17],
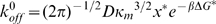
Here, 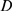 is the effective diffusion coefficient, 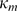 is the molecular spring constant, 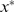 is the distance along the free energy well from the minimum to bond rupture, and 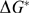 is the minimum bond potential energy. This value of 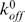 was then used to calculate 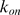 for all bonds using the energy relation
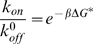
For already existing bonds between cell and virion, 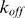 values were calculated using the relation
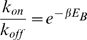
where the bond potential energy, 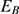, was calculated using a parabolic approximation of the Lennard-Jones potential,
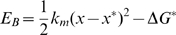
Here, 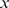 is the distance along the energy potential calculated by subtracting the length of the proteins involved in the bond from the shortest distance between the location of the proteins on the plasma membrane and the virion.

The total probability of transitioning out of the current state was equal to the sum of all forward rates for each possible destination state:
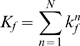
The specific destination state of the system was determined by a pseudo random number generator (PRNG). Briefly, once all possible states available to the current state are determined and their forward rates are calculated, the probability (or rate constant) describing the likelihood that the system transitions away from the current state is the total sum of all forward rates, 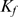. To determine which of these possible states is the next destination state, the PRNG yields a random number, 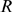, uniformly distributed between 0 and 1. 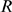 is multiplied by 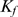 resulting in a random position between 0 and 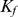, which corresponds to a particular adjacent state. The system is subsequently updated, newly available states are determined, and their new forward rate constants are calculated according to the imposed changes (e.g. if a gp120-CD4 bond breaks, new 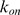 rates are calculated for the newly free gp120 and CD4 molecules). This process was repeated, updating each new state sequentially. Energy changes that governed the evolution of the system included those of individual gp120-CD4 bonds, gp120-CCR5 bonds, and the deformation of the plasma membrane with a specified elastic modulus and surface tension.

The fluctuations of the membrane, diffusion of the receptors and the diffusion of the virus are described by Fokker-Planck equations. The simulation methodology is described in Atilgan *et al.* [18]. The total free energy of the system, 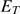, is given by
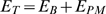
where 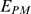 is the free energy of the plasma membrane calculated using the Canham-Helfrich form [19], [20],
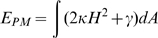
Here, 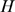 is the mean curvature of the membrane, 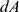 is the local area, 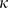 is the elastic modulus and 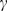 is the surface tension of the membrane. It should be noted that electrostatic interactions between protein pairs not bound together and between the viral and cellular membranes were not included in the computation of the energy for a given state. In addition, we simplified our model by assuming that the concentration of the local actin filament network beneath the cellular membrane is sufficiently low so as to not dictate plasma membrane deformation [21].

The time elapsed as the system stepped from one state to another was also calculated and used to determine the total time elapsed during the simulation, starting at 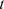 = 0s. The duration of each time step was calculated using the equation
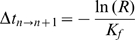
Here, 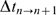 is the time step from the 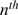 to the 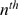
+1 state and 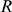 is the uniformly distributed random number between 0 and 1 provided by the PRNG.

The plasma membrane was initially defined as a completely flat surface. To simulate a more realistic interaction between cell and virion, the plasma membrane was allowed to evolve during the initialization of the system without the ability to form productive bonds with the virion above it. After this brief initialization (2×10^6^ sequential iterations), the simulation of receptor-mediated viral adhesion to the cell surface was allowed to begin. Surface proteins on the plasma membrane were randomly distributed during the initialization according to the PRNG and concentrations of diffusing, unbound proteins were kept constant throughout the simulation. Proteins on the viral surface were either evenly spaced over the entire particle as previously described [22], or randomly distributed according to the PRNG using a random zenith, θ, between 0 and π according to the probability density distribution 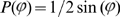, and random azimuth, 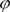, uniformly distributed between 0 and 2π.

Throughout the simulation, proteins had a finite volume, so that other proteins were not allowed to diffuse through one another on either the viral surface or the plasma membrane. In addition, the plasma membrane and virion could not occupy the same space. If a physical obstacle was encountered, the forward rate for that adjacent state was set equal to 0. The actual lengths of gp120, CD4 and CCR5 molecules were also used when calculating bond interaction distances and free energies (Table 1). Lastly, gp120 trimers located on the viral surface were capable of binding up to three CD4 molecules and three CCR5 molecules at a time. As stated earlier, CCR5 adhesion to gp120 in contingent on a previously existing gp120-CD4 bond. In our system, gp120 trimers were not allowed to bind CCR5 unless that trimer was already involved in a gp120-CD4 bond. However, the number of CCR5 bonds was not allowed to exceed the number of CD4 bonds formed with a single gp120 spike, i.e. no synergistic effect was imposed throughout a gp120 trimer that would allow a single CD4 adhesion to promote multiple CCR5 bonds. For a more detailed explanation of the modeling algorithm see Text S1.

**Table 1:** System parameters used to simulate viral adhesion dynamics.

Parameter	Value	Unit	Ref.
Plasma membrane size	200×200	nm	
Plasma membrane D	1×10^6^	nm^2^/s	
Plasma membrane κ	20 and 100	k_b_T	[35]
Plasma membrane γ	0.005	k_b_T/nm^2^	[21]
Virion radius	50	nm	[36]
Virion D	100	nm^2^/s	[3]
Number gp120 trimers	7,11,14,15,20		[3]
gp120 D	12	nm	[3]
Length gp120	8	nm	[37]
CD4/CCR5 ratio	10−1		[29]
CD4 D	5.0E5	nm^2^/s	[38]
CCR5 D	4.5×10^5^	nm^2^/s	[38]
Bond angle limit	40	°	
**CD4-gp120 Bond**			
K_off_ Initial	165	1/s	[14]
K_off_ Unstable	2239	1/s	[14]
X^‡^	0.75	nm	[14]
ΔG^‡^	−6.7	k_b_T	[14]
κ_m_	25	k_b_T/nm	[14]
Length CD4	6.2	nm	[39]
**CCR5-gp120 Bond**			
K_off_	185	1/s	[14]
X^‡^	0.36	nm	[14]
ΔG^‡^	−7.6	k_b_T	[14]
κ_m_	118	k_b_T/nm	[14]
Length CCR5	3.5	nm	[40]

The system parameters including their units and reference are listed. In addition, the range of values explored for system parameters is also listed if applicable.

## Results

### Organization of the virion-cell junction for fixed orientation of gp120 trimers on the viral surface

**Figure 2:**
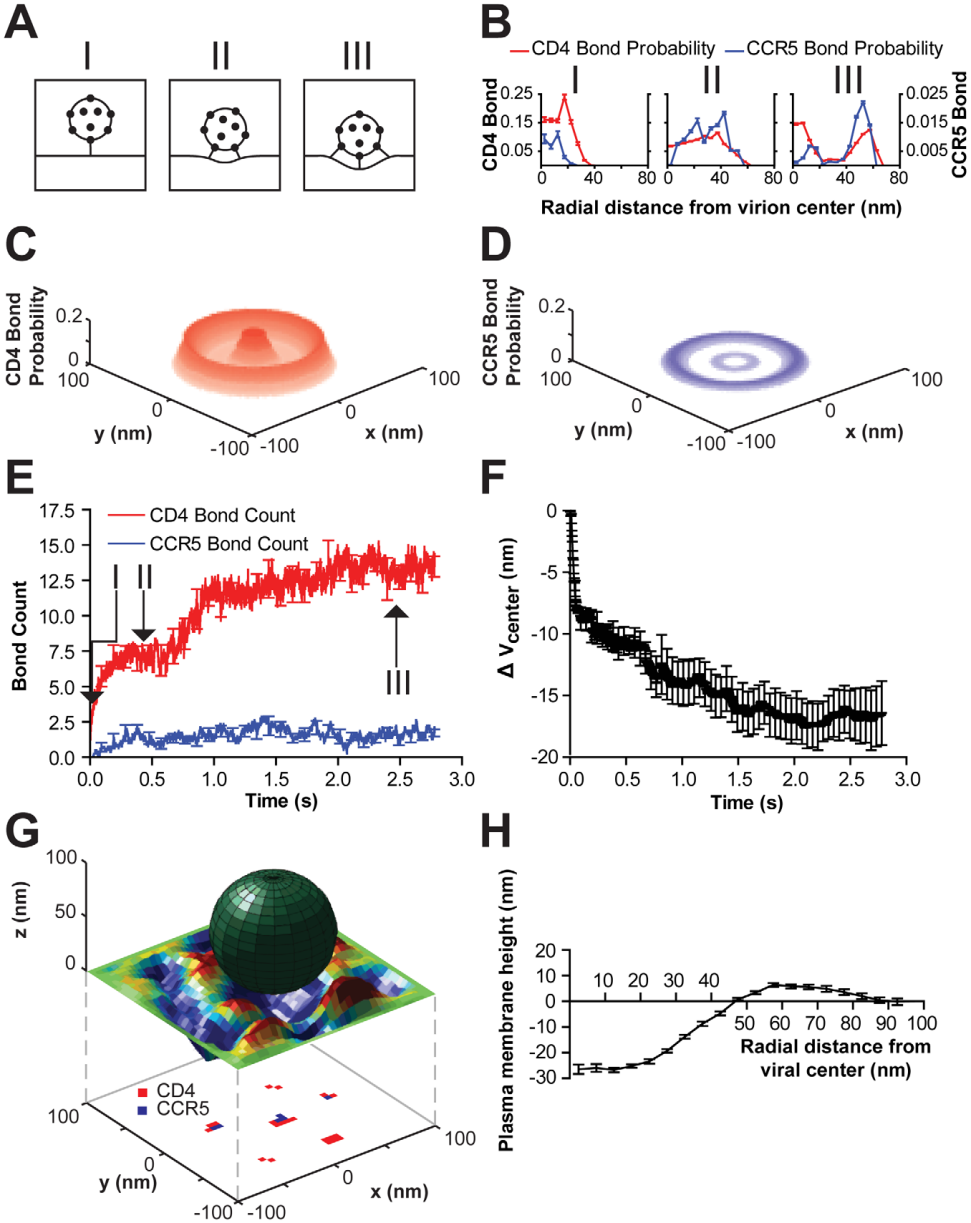
Organization of proteins within the viral junction for fixed, evenly distributed viral trimers. (**A**) Schematic describing the three main phases through which the organization of intermolecular bonds between a virion and cellular receptors on the plasma membrane evolves. (**B**) Probability distributions of cellular receptors bound to the virion for the three organizational phases as a function of the radial distance from the viral center. The steady state (phase III) bond probability distributions for bound CD4 and CCR5 receptors were time-averaged over the last 2.5×10^8^ iterations (Δt∼0.5s). Phase I and II probability distributions were time-averaged over 2×10^7^ iterations (Δt∼0.02s). (**C and D**) Three-dimensional projections of the steady state (phase III) probability distributions for bound CD4 (C) and CCR5 (D) receptors (origin is the viral center). (**E**) The number of bound CD4 (red) and CCR5 (blue) bonds in the viral junction increase with time as the system progresses through the three distinct phases (I, II, and III indicated by arrows) toward steady state. Error bars are depicted every 5×10^7^ iterations. (**F**) As the plasma membrane deforms and bonds begin to form, the height of the virion (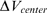, the change in position of viral center from initial cell contact) decreases, approaching a steady state. (**G**) A typical realization of the viral junction including a rigid sphere above a flexible plasma membrane with CD4/CCR5 bond locations projected beneath. (**H**) The plasma membrane of the system deforms and approaches the curvature of the partially engulfed virion at steady state. Shown here is a radial profile of the plasma membrane i.e., its vertical displacement as a function of the radial distance from the viral center. Simulations were performed with 15 gp120 trimers per virion and eight such simulations were averaged to produce the results illustrated here.

First, we studied the development of viral-cell adhesion with a viral gp120 organization in which protein trimers were evenly distributed over the viral surface and were not allowed to move. We observed that as the system progressed towards steady state, the gp120-CD4 bond probability distribution displayed three distinct phases of organization (Fig. 2A).

The system quickly transitioned from single gp120 trimers bound to the plasma membrane, which we call phase I, to a second state through rotation and translocation, which allowed multiple gp120 trimers to bind to cellular receptors and produced a single broad node of bond probability formation, which we call phase II (Fig. 2B). Accordingly, upon initial physical contact between the cell and virion, the gp120-CD4 bond probability distribution displayed a single maximum near the viral center (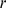
∼0nm). This is a result of initial gp120-CD4 bonds occurring most preferably at the closest point on the virion to the plasma membrane (Fig. 2B). The shift of the bond probability maximum from the viral center is the result of averaging the initial bond locations of multiple simulations (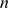 = 8) where the viral particle is binding to a non-uniform plasma membrane surface. At the initial time of contact the closest point on the cellular membrane is not always presented to the virion directly at 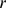 = 0nm. Phase II resulted in a broadening of the CD4 bond probability distribution, during which its maximum shifted to a distance 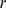 = 37nm from the center (radius of the virion, 50 nm), i.e. CD4 receptors participating in the viral junction organized into a ring-like structure or corona (Fig. 2B). CCR5 bonds also organized into a corona, with a spatial distribution similar to that of CD4 but containing much fewer bonds (Fig. 2B).

Through continued rotation and translocation of the virion, the interfacial region between cell and virion further evolved to develop a central, “anchor” gp120 spike surrounded by trimers bound to adjacent cellular receptors. This configuration produced a bimodal bond probability distribution of bound cellular receptors, which we call phase III (Fig. 2, A–D). While the organization of the adhesive junction between the cell and virion developed, the flexible plasma membrane spontaneously deformed and engulfed the virion. The deformation of the plasma membrane increased the cell surface area which was close enough to the virion to allow further receptor binding to gp120 trimers, thereby increasing the total bond number (Fig. 2E). The time of formation of an organized viral junction (Fig. 2, C and D) remained fairly constant over multiple simulations with different (random) initial states indicating that the final organization and number of bonds in the viral junction were relatively independent of the initial positions of the cellular membrane, virion, and receptors.

**Figure 3:**
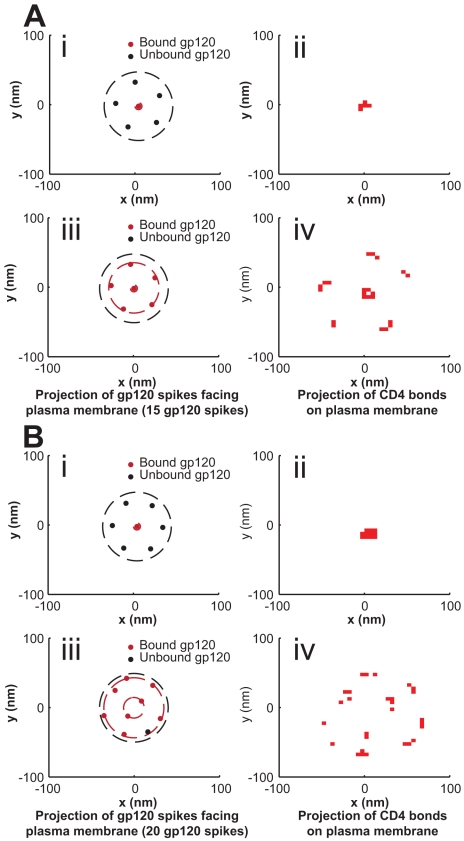
Example organization of the gp120 trimers facing the plasma membrane and resulting CD4 bond organization. (**A and B**) Initial organization of bound and unbound gp120 trimers on the viral surface facing the plasma membrane (i) and the resulting CD4 bond organization on the cell surface (ii). Final organization of bound and unbound gp120 trimers on the viral surface facing the plasma membrane (iii) and the resulting CD4 bond organization on the cell surface (iv). Simulations were conducted using either 15 (A) or 20 gp120 trimers (B) per virion. The orientation used here is that of looking down along the y-axis through the virion (represented by the dashed circle) onto the plasma membrane.

The virion formed a stable adhesion interface and progressively increased the number of bonds. Simultaneously, the position of the virion above the membrane decreased as the membrane deformed to engulf the virion with time. Here, the change in the vertical height of the virion from its position at initial cell contact (i.e. the depth of virion engulfment) is referred to by 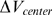, and the evolution of 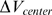 as adhesion progresses from Phase I to Phase III is illustrated in Fig 2F. Interestingly, after CD4 and CCR5 bond probabilities had reached steady states, the depth of engulfment of the virion continued to increase until the plasma membrane reached an equilibrium deformation (Fig. 2, G and H). Again, there are two counteracting “forces” that dictate the direction in which the organization of the virion-cell interface progresses. The first force is the energetically favorable formation of bimolecular bonds between proteins on the viral and cell surfaces; the second force is the energetically unfavorable deformation of the plasma membrane. The plasma membrane may be maintained in an unfavorable, deformed position if it is permissive of an increase in bond number (Fig. 2H). Fixed gp120 units on the viral surface forced the virion to maximize bond formation by rotating and laterally moving the virion so as to minimally deform the plasma membrane, while recruiting new receptors to bind. Eventually, the depth of engulfment of the virion and the radial profile of the plasma membrane stabilized at heights that no longer exposed new gp120 units to cellular receptors on the plasma membrane. The organization of spatially fixed gp120 molecules on the viral surface regulates the organization of bonds between cell and virion (Fig. 3).

Together these results suggest two important findings: (i) CD4-gp120 bimolecular bonds can be highly organized in the interfacial region between the cell and virus; (ii) the receptor organization is dynamic: initially a peak forms at the center, followed by a corona or bull's eye pattern of virus-cell bonds develops, and finally a peak and a corona co-exist while the plasma membrane deforms and engulfs the virion (Fig. 2, C and D).

In the following, we conduct simulations over a wide range of parameters to investigate the effects of viral protein organization, receptor concentration, plasma membrane rigidity, and overall bond stability between the cell and virion, on the organization of cellular receptors.

### Effect of gp120 configurations on the organization of the viral junction

**Figure 4:**
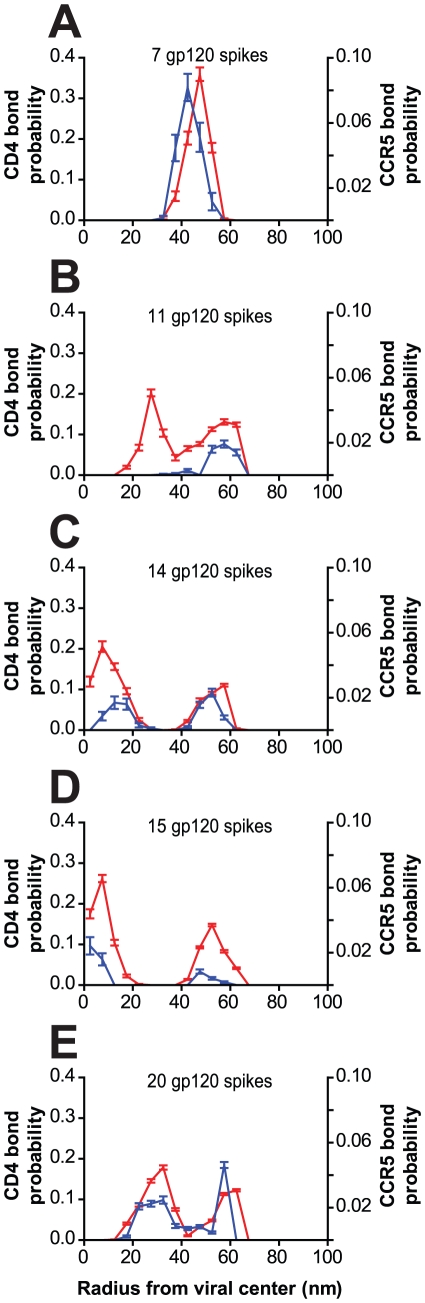
Bond profiles of bound cellular receptors for different numbers of evenly distributed viral trimers. (**A–E**) Example 2-D CD4/CCR5 bond probability distributions produced by viral particles with different numbers of gp120 trimers evenly distributed on the viral surface adhering to the cell membrane. As the number of gp120 trimers on the viral particles increases, the complexity of the bond probability distribution also increases.

Recent studies suggest that the increased density of gp120 on the surface of viral particles could increase infection [3], [23], [24]. Therefore, we studied the effect of gp120 density (at fixed positions on the viral surface) on the organization of the viral junction over the physiological range of 7–20 gp120 trimers per virion [3]. We found that, when viral particles contained few gp120 (7–9 trimers), organized viral junctions did not form and little plasma membrane deformation occurred (Fig. 4). For an increased number of gp120 trimers per virion (14–20 trimers), the distance between adjacent gp120 trimers was sufficiently reduced that spontaneous deformations in the plasma membrane could result in an increase in the number of bonds (Fig. 4). The number of bound CD4 and CCR5 bonds correlated directly with the number of gp120 trimers on the viral surface (data not shown), indicating that reduced gp120 density result in fewer virus-cell bonds, a less dynamic protein organization within the viral junction, and greatly reduced plasma membrane deformation.

### Effect of gp120 diffusion on the viral surface on the organization of the viral junction

**Figure 5:**
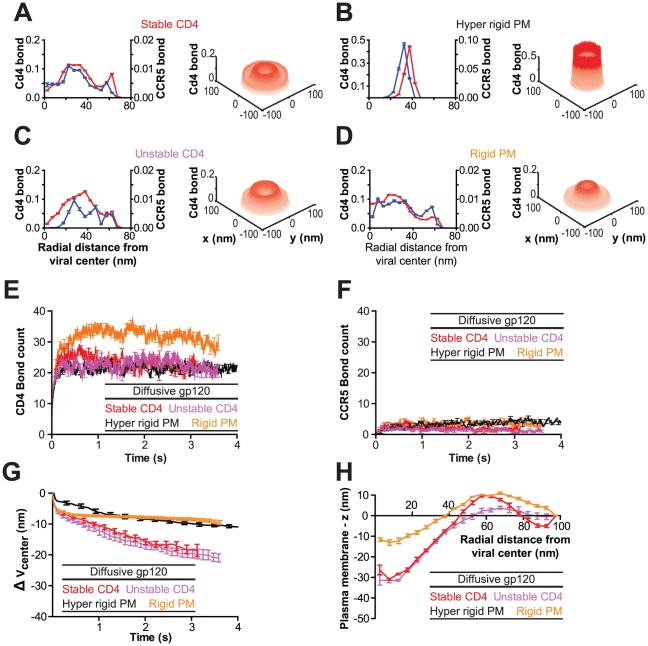
The organization of the viral junction depends on the rigidity of the plasma membrane and the stability of the gp120-CD4 bond – the case of gp120 trimers diffusing on the viral surface. (**A–D**) Two- and three-dimensional bond probability distributions of bound receptors when gp120 trimers are allowed to diffuse on the viral surface. The following illustrative cases for the organization of the viral junction are shown: (A) stable CD4/CCR5 molecules (Stable CD4) with plasma membrane rigidity of κ = 20 k_b_T/nm, (B) stable CD4/CCR5 molecules on a completely flat surface (Hyper rigid PM), (C) CD4 molecules with an induced instability (Unstable CD4; see text for details) and plasma membrane rigidity of κ = 20 k_b_T/nm and (D) stable CD4/CCR5 molecules on a rigid plasma membrane (Rigid PM, κ = 100 k_b_T/nm). Bond probability distributions were time-averaged over the last 2×10^7^ iterations (Δt∼0.015s, n = 8). (**E and F**) The number of bound CD4 (E) and CCR5 (F) bonds increase with time and approach steady states. Conditions correspond to panels A–D, as indicated. (**G**) The engulfment of the virion can be measured by its change in height where 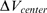 is the change in position of viral center from initial cell contact. Conditions correspond to panels A–D, as indicated. (**H**) The steady state profile of the plasma membrane (the x-axis is measured in nm from the viral center, while the y-axis is the average variation of the membrane height from initialization of the system). Conditions correspond to panels A–D, as indicated. Simulations were performed with 15 gp120 trimers per virion and eight such simulations were averaged to produce the results displayed here.

Recent work suggests that gp120 units may diffuse on the viral surface [25]. To determine whether diffusing gp120 trimers on the viral surface could also produce an organized viral junction, we conducted simulations allowing gp120 trimers to diffuse freely on the viral membrane (Fig. 5). The presence of freely diffusing gp120 trimers allowed for the formation of three times as many CD4 bonds than in the fixed gp120 case (Figs. 2E and 5E). These bonds also formed 10 times faster than for virions with fixed gp120 positions. While the number of CD4 bonds increased beyond the steady state value of the fixed case, the number of CCR5 bonds did not change significantly from the fixed case (Figs. 2E and 5F).

Initially the CD4 bond probability profile in the organized viral junction was similar to that of the case of spatially fixed gp120 units. Then, the maximum of the bond probability grew outward resulting in a local maximum at a stable distance 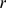
∼27nm from the center (Fig. 5A). After a slight delay, the CCR5 bond probability grew in a similar manner, but was dwarfed by the probability of forming CD4 bonds (Fig. 5A). The probability distribution of bound cellular receptors did not feature a single peak directly beneath the center of the virion because of the deformation of the plasma membrane. The resulting curvature of the plasma membrane while induced by the virion, did not exactly match the virion curvature. These mismatched curvatures resulted in only a fraction of the area directly under the virion to be close enough to mediate the formation of bonds between viral and cellular receptors (Fig. 5, G and H, Stable CD4 and Rigid PM). These mismatched curvatures did not result from specific random starting conditions, distributions shown here result from eight independent simulations. As in the fixed gp120 case, virions with freely diffusing gp120 induced membrane deformation and resulted in viron engulfment (Fig. 5, G and H). Ultimately, membrane deformation resulted in a depth of virion engulfment of 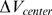 = 22nm. However, while the number of bonds increased much faster than in the fixed gp120 case, plasma membrane deformation and virion engulfment occurred at rates similar to those in the fixed gp120 case (Figs. 2F and 5G).

**Figure 6:**
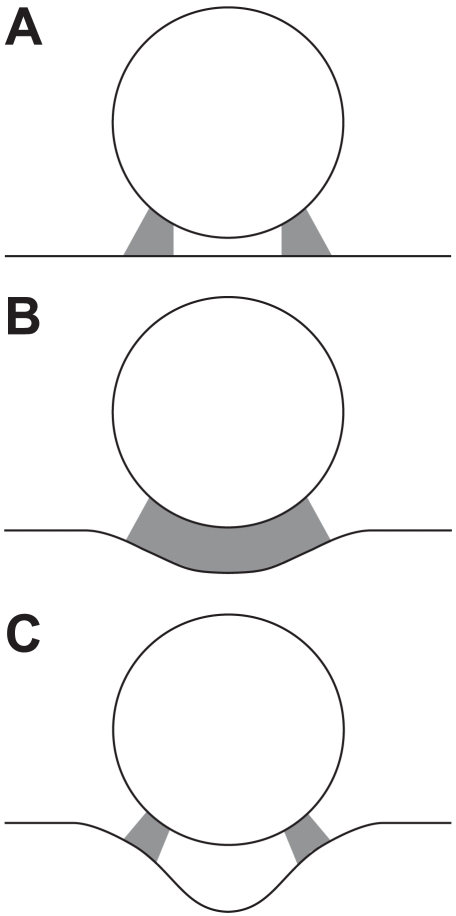
Cellular membrane rigidity will determine the characteristic deformation of the membrane and directly affect the adhesion competent area on the cell. (**A–C**) Schematic of area allowing for bond formation between a viral particle and cellular receptors located on a completely rigid plasma membrane (A), a flexible plasma membrane (B), or a comparatively more flexible cellular membrane (C). The shaded areas here illustrate the adhesion competent area (i.e. at an optimal distance for bonds to form) between the virion and the cell.

The location of bound cellular receptors corresponds to the shortest distance between the virion and the plasma membrane. When the plasma membrane is completely flat (*κ* = ∞), the virion is brought in close proximity to the cell to maximize the adhesion competent viral surface area (Fig. 6A), cellular receptors at 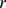 = 0 nm cannot bind the virion due to lack of space, resulting in a corona (Fig. 5, B, G, and H, hyper rigid PM). When the rigidity of the plasma membrane is relatively low (*κ* = 20 k_b_T/nm), the maximum in the probability of bound receptors away from 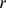 = 0nm, i.e. a corona of bound receptors is formed. When the rigidity of the plasma membrane is increased (*κ* = 100 k_b_T/nm; Fig. 5, D and H, Rigid PM), the steady state deformation of the plasma membrane is reduced and the maximum probability of bound receptors is shifted toward the radial center. Simple geometric analogies for the soft and rigid plasma membrane case are a sphere sitting in the bottom of a cone compared to a sphere sitting at the bottom of a larger sphere, respectively (Fig. 6, B and C).

The slight decrease in bond number over time for the case of diffusive gp120 (Fig. 5E, Stable CD4 and Rigid PM) also results from the membrane deformation. Initially, bond number increases rapidly because diffusive gp120 molecules can concentrate between the virion and the plasma membrane. Bond number decreases slowly when the plasma membrane deforms, limiting the adhesion-competent area for cellular receptors to occupy (Fig. 6). Taken together these results suggest that the potential ability of gp120 molecules to diffuse in the viral membrane has a qualitative effect on the type of organization of the virus-cell interface and a quantitative effect on the number of bound receptors in that interface.

We note that a virion with diffusing gp120 trimers produces patterns of bound receptors similar to those recently reported using electron tomography [25], especially those produced by CD4 receptors with biphasic stability (Fig. 5C, Unstable CD4; see more details below and in the Discussion section).

### Effect of gp120 distribution on the organization of the viral junction

Previous work suggests that gp120 trimers are unevenly distributed on the viral surface [3], [25] and that these trimers may form fixed clusters [3]. We examined whether the formation of an organized viral junction underneath the virion containing a corona of bonds at an intermediate radial distance required uniformly distributed gp120 trimers by examining viral adhesion governed by randomly distributed gp120 trimers on the viral surface. Frequently, interfaces developed by these virions contained randomly clustered gp120 units which resulted in complex bond distributions similar to that produced with evenly placed gp120 (Fig. S1). However, the distance between the concentric rings of maximum bond probability depended on the spacing of gp120 on the viral surface and varied from one simulation to the next.

This double-corona viral junction was a direct result of the non-uniform gp120 spike distribution on the viral surface. The interaction between local viral gp120 organization and receptors on the plasma membrane resulted in the progression of bond organization through distinct phases similar to the case of evenly distributed gp120 (Fig. 2B and Fig. S1). The virion first made contact with the plasma membrane, rotated, and as the deformation of the plasma membrane continued, a bimodal bond probability was established as virion rotation presented gp120 trimers previously unattainable to the receptors. The distance between gp120 trimers ultimately determined the distance between the nodes of the bond probability distribution (Fig. S1). Indeed, other simulations resulted in distributions that resembled phase II organization, i.e. a single-maximum bond probabilities single (Fig. 2A, Fig. S1). Unbound gp120 trimers that were spaced too far away on the viral surface for the virion to successfully rotate and expose to the cellular receptors resulted in halting the progression of bond organization at a single, off center probability maxima (Fig. S1). Together these results suggest that the final organization of CD4 receptors bound to fixed gp120 at the virus-cell interface before viral entry depends on the spatial organization of gp120 molecules on the surface of virions.

### Effect of plasma membrane rigidity on the organization of the viral junction

**Figure 7:**
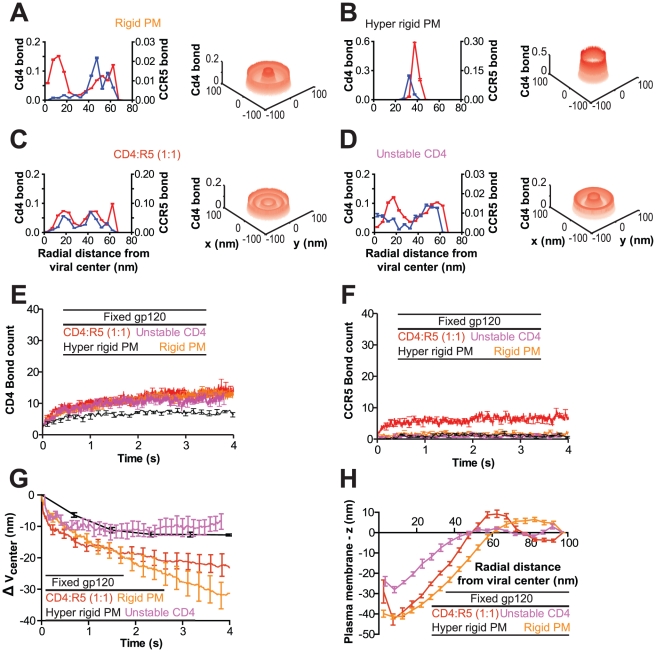
The organization of the viral junction depends on the rigidity of the plasma membrane, the stability of the gp120-CD4 bond, and the concentrations of cellular receptors – the case of fixed gp120 trimers. (**A–D**) Two- and three-dimensional bond probability distributions of bound receptors CD4 and CCR5 for a fixed (evenly spaced) configuration of gp120 trimers. The following illustrative cases are shown: (A) stable CD4/CCR5 molecules (Stable CD4) with plasma membrane rigidity of, κ = 20 k_b_T/nm, (B) stable CD4/CCR5 molecules on a completely flat surface (Hyper rigid PM), (C) CD4 molecules with an induced instability (Unstable CD4; see text for details) and plasma membrane rigidity of, κ = 20 k_b_T/nm,, and (D) stable CD4/CCR5 molecules on a rigid plasma membrane (Rigid PM, κ = 100 k_b_T/nm). Bond probability distributions were time-averaged over the last 2×10^7^ iterations (Δt∼0.015s, n = 8). (**E and F**) The number of CD4 (E) and CCR5 bonds (F) increase with time and approach a steady state. Conditions correspond to panels A–D, as indicated. (**G**) The engulfment of the virion can be measured by its change in height where 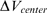 is the change in position of viral center from initial cell contact. Conditions correspond to panels A–D, as indicated. (**H**) The steady state profile of the plasma membrane (the x-axis is measured in nm from the viral center, while the y-axis is the average variation of the membrane height from initialization of the system). Conditions correspond to panels A–D, as indicated. Simulations were performed with 15 gp120 trimers per virion and eight such simulations were averaged to produce the results displayed here.

The depletion of cholesterol from cellular membranes may significantly inhibit HIV-1 infection [26] and patients treated with cholesterol lowering statins seem to present decreased viral loads [27]. Aside from affecting subcellular pathways activated by statins, the depletion of cholesterol can dramatically affect the mechanical stiffness of the plasma membrane [28]. Previous work has demonstrated that varying cholesterol levels have a direct effect on the deformability of lipid vesicles [28]. We found that the plasma membrane rigidity critically influenced the spatial organization within the viral junction (Figs. 5D and 7, A and B).

Cells with a completely rigid plasma membrane (*κ* = ∞) coupled with evenly distributed gp120 units never progressed beyond the second phase of adhesion/bond formation described above (Fig. 7B). This completely rigid plasma membrane resulted in slightly fewer CD4 bonds than in viral junctions involving cells with a flexible membrane and virions with fixed gp120 trimers (Fig. 7E). A fivefold increase in membrane rigidity (*κ* = 100 k_b_T/nm *vs.* 20 k_b_T/nm) did not significantly change the steady state number of bonds in the viral junction (Fig. 7E, Rigid PM) and viral junctions typically took approximately twice as long to progress beyond the second phase of adhesion. Moreover, a fivefold increase in membrane rigidity resulted in a little more than double the depth of virion engulfment (Fig. 7G, Rigid PM).

For systems containing diffusing gp120 trimers adhering to receptors on a more rigid membrane, the virion depth of engulfment quickly stabilized at almost half the depth observed with a more flexible plasma membrane (Fig. 5G). This was accomplished without a significant change in the numbers CCR5 bonds (Fig. 5H). However, a more rigid membrane in conjunction with diffusive gp120 resulted in increased CD4 bond numbers and a higher probability of bond towards the center, 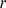 = 0 nm (Fig. 5, D and E, Rigid PM). Together these results suggest that the mechanical properties of the plasma membrane can affect the organization and distribution of bonds within the viral junction. If organized virion-cell bonds are indeed important for successful infection, then the effect that cholesterol concentration has on the mechanical properties of the plasma membrane may contribute to its effect on HIV-1 infection.

### Effect of CD4∶CCR5 molar ratio on the organization of the viral junction

The concentration of CD4+ T cells harvested from the blood and lymph node tissue of infected patients correlates with infection and the ratio of [CCR5]∶[CD4] [29]; infection will correlate more to CD4 expression when CCR5 is expressed in limiting amounts and *vice versa* [30]. Our results indicate that the steady state number of CCR5 bonds in the viral junction depended most critically on the concentration of CCR5, and did not strongly depend on the mechanical stiffness of the plasma membrane or the number, mobility, and the organization of gp120 on the virions (Fig. 5F and 7F).

For fixed gp120 trimers on the virion, a tenfold increase in CCR5 concentration produced a twofold increase in steady state number of CCR5 bonds in the viral junction (Figs. 2E and 7F, for 10∶1 and 1∶1 [CD4]∶[CCR5] molar ratios, respectively). When CCR5 concentration was increased to the same level as CD4, the physical hindrance of additional bound proteins located in an area of comparable size became more of a determining factor for protein organization than when CCR5 was present at a lower concentration. For example, increasing the CCR5 concentration resulted in a CD4 bond probability distribution containing three local maxima (Fig. 7C). The increased number of CCR5 molecules, coupled with the increased stiffness and decreased length of the CCR5 bond quickly resulted in a virion with comparably little rotational freedom. The virion was bound by so many more CCR5 proteins than in any other simulation described here, that it had a much greater resistance to rotation and translocation so to minimize bond free energy (because of the higher stiffness of CCR5 bonds).

Figures 7, G and H, compare the depths of virion engulfment and the plasma membrane profiles for increased CCR5 concentration, [CD4]∶[CCR5] = 1∶1. While the plasma membrane has a similar z-position for a more rigid membrane (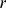
∼10nm away from viral center), the virion itself is sitting almost 10 nm higher than with a rigid membrane. This increased resistance ultimately results in phase III organization of bonds, however the increased CCR5 bonds at larger radii produced a CD4 bond probability distribution with three local maxima (Fig. 7C). The two outward CD4 bond probability maxima (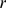
∼45nm and 65nm) could have actually formed a single maximum, were it not for the steric hindrance of the shorter CCR5 bond coupled with the local plasma membrane deformation. The shorter CCR5 bonds concentrate in a small area of the plasma membrane and surrounded by the longer CD4 bonds which are able to diffuse over a larger area. Unable to occupy the same space, two local CD4 probability maxima flanking the outward CCR5 bond probability maxima were formed.

### Effect of CD4-gp120 bond instability on the organization of the viral junction

We recently demonstrated that the presence of CCR5 could result in a decrease of CD4-gp120 bond stability [14]. Therefore, we examined the effect of CD4-gp120 bond instability on viral junction formation and organization. For fixed gp120, CD4-gp120 bond instability resulted in fewer CD4 bonds between the cell and virus, averaging 8 at long time scales, as well as a decrease in average CCR5 bond number to practically zero (Fig. 7, E and F). The CD4 bond probability distribution was noticeably bare at the center of the virion-cell interface region compared to the stable CD4 bond case, indicating a slight difference in the third phase of organization (Fig. 7D).

Initial CD4 bonds were formed and increased in number similarly to the previous cases. However, when CCR5 bonds began to form and the CD4 bonds became unstable. CD4-gp120 bonds broke and the CD4 molecules diffused away, leaving only the CCR5 bond between the cell and virus. Ultimately, the last CCR5 bond broke and could not reform as no CD4 bonds were sufficiently close to initiate the gp120 conformation change. This production of CCR5 bonds and destruction of local CD4 bonds continued while the viral-cell interface as a whole maintained a constant number of bonds (Fig. 7, E and G), forming a globally stable adhesion interface (Fig. 7D). Interestingly, imposing a biphasic gp120-CD4 instability while also allowing gp120 trimers to diffuse on the viral surface most accurately recreated the bond organization previously observed using electron tomography (Fig. 5C).

## Discussion

Our computational results suggest that a viral particle can induce the formation of a highly organized ring-like ultrastructure of cell receptors bound to viral proteins, which we termed the viral junction. Our model involves biochemical (e.g. binding constants) and biophysical parameters (e.g. membrane stiffness) that have previously been measured. The diffusion rate constants of the plasma membrane and gp120 on the viral surface were assumed. However, these two unknown constants only set the rate of formation of a viral junction, not its steady state organization. Results from the model suggest that the formation of an organized viral junction is robust against relatively large variations in receptor concentrations, virion properties, physical properties of the plasma membrane, and dynamic properties of virus-cell bimolecular bonds.

The simulations revealed that several factors contribute to the organization of bonds on the flexible membrane. The ability of CD4 and CCR5 molecules to diffuse while bound to gp120 contributes to the organization of the viral junction by increasing bond formation while decreasing plasma membrane deformation. For fixed gp120 (i.e. unable to diffuse on the viral surface), the organization of the viral junction primarily depends on the gp120 distribution on the viral surface. While for the diffusive gp120 case, the organization of the viral junction primarily depends on the deformability of the plasma membrane. The longer and more flexible CD4 bond compared to the CCR5 bond, coupled with the local deformation of the plasma membrane, often result in different bond organization for these two receptors.

Our computational model suggests that the mechanical properties of the plasma membrane work in concert with viral gp120 organization to organize cellular receptors at the virion-cell interface. Changes in the stiffness of the plasma membrane, which could be mediated by changes in cholesterol content [26], affect the properties of the viral junction, including the total number of bonds between cell and virion. The virion-cell bonds work with the plasma membrane to reduce the overall potential energy of the system by two mechanisms. First, an energetically unstable bond forms where the plasma membrane is not locally deformed and the membrane is subsequently deformed to stabilize the bond. Second, the membrane spontaneously deforms to an unfavorable configuration and before it can relax a receptor forms a bond that maintains the deformation of the plasma membrane. The finite deformability of the plasma membrane limits how much the membrane can spontaneously deform without forming new bonds. By spacing gp120 trimers too far away on the viral surface for the plasma membrane receptors to spontaneously encounter them, the distribution of gp120 dictates the extent of plasma membrane deformation.

Recently it has been suggested that endocytosis plays an important role in HIV-1 infection [4]. The formation of an organized viral junction, which is computationally described here within a small 200×200nm area, could occur anywhere from the plasma membrane to within an endocytic vesicle.

Previous theoretical work using thermodynamic steady states predicted that an increase in membrane rigidity would decrease the number of bonds between cell and virion [21]. However, this conclusion was reached assuming a uniformly binding viral surface. Here, discrete locations of adherent gp120 trimers reveal that the point at which the plasma membrane is unable to bind and continue deformation is dependent on the distance at which unbound gp120 units are spaced.

A computational model of how CD4 receptor organization on a planer surface responds to binding gp120 trimers has previously been introduced [31]. This earlier work focused on the rate at which the gp120 molecules of a trimeric spike become bound to CD4 as a function of gp120 density and CD4 diffusion coefficient. While this work predicts an increase in local CD4 concentration under the virion due to bond formation, it does not appear to display receptor organizations similar to those predicted by our work. This difference may stem from this earlier works use of a rigid plane to simulate the cellular membrane and the absence of co-receptor adhesion. Here, we employ a flexible membrane, which we demonstrate plays a critical role in the organization of the viral junction. The addition of coreceptor adhesion also offered insight into the roles that CD4 and CCR5 bond micromechanics play in the formation of the viral junction. Lastly, we had the advantage of using experimentally measured kinetic and micromechanical values for gp120-CD4 bonds.

Recent reports suggest that gp120 could be partially disorganized or diffuse on the viral surface [25] and that viral infection correlates with gp120 concentration [3], [23]. Therefore, we studied the effects of diffusing *vs.* non-diffusing gp120, as well as gp120 density on the formation, organization, and dynamics of the viral junction. Viral particles with diffusive gp120 organized cellular receptors into a distinct corona to maximize the number of bonds between the cell and virus (Fig. 5, A–D). Sougrat *et al.* suggest that a virion adhering to a cell induces a similar formation of proteins between the cell and virus, called the ‘entry claw’, with comparatively little gp120 elsewhere on the virion. It was suggested that this concentration gradient in gp120 along the viral surface is best explained by the ability for gp120 to diffuse on the surface. The conditions that best reproduced the bond organization observed using electron tomography is a combination of diffusive gp120 trimers with an imposed biphasic stability on the gp120-CD4 bond, suggesting that gp120 are not permanently organized on the viral surface. However, the gp120 gradient observed by Sougrat *et al.* can also be explained by the possibility that virions with clustered gp120 trimers have preferential binding as well as the possibility that gp120 trimers could be cleaved from the virion during sample preparation for electron tomography. Therefore, in addition to the diffuse case, we also considered the effect of gp120 configurations on viral junction organization.

An experimental test of our computational predictions is challenging given the small sizes of the virion and associated viral junction. However, the advent of super resolution microscopy approaches, such as photoactivated localization microscopy (PALM) [32], could help determine the organization of the viral junction through co-labeling of gp120 and receptors CD4 and CCR5. Combined immunolabeling and cryo-electron microscopy (EM) or tomography could also help assess the organization of the viral junction; however EM often creates artifacts especially when visualizing the plasma membrane.

If we assume that certain bond configurations result in enhanced infection, then the subpopulation of infectious viral particles not only depends on gp120 density, but also gp120 organization on the viral surface. A viral population containing particles equipped with a functional gp120 organization (i.e. with relatively clustered gp120 on the viral surface) could lead to the formation of an organized viral junction, while those with dysfunctional organizations (with relatively distant gp120), may be unable to infect cells. Since the formation of a viral junction depends on receptor density and the mechanical properties of plasma membrane, viral junction formation could be cell type-specific. One might speculate from the spectrum of mechanical properties exhibited by different cell types as well as the distribution of viral particle size and gp120 concentration that a subset of viral particles might preferentially infect one cell type while another subset of particles could preferentially infect another cell type.

Although the formation of the viral junction would occur at the much smaller length scales than that of a whole cell, the organization of the viral junction is reminiscent of the immunological synapse. It is therefore tempting to speculate on the possible signaling function of the viral junction. For instance, gp120 binding induces assembly of a local actin network and coreceptor binding induces disassembly of actin filaments [33], [34]. By analogy to the better-characterized immunological synapse, we speculate that part of this signaling function could be related to the complex evolution of an organized viral junction by relying on one organizational phase between receptor and coreceptor for an initial signaling event and a subsequent organizational phase for a secondary signaling event.

## Supporting Information

Figure S1 Bond profiles of bound cellular receptors for gp120 trimers randomly distributed on the viral surface. (A–F) Example 2-D CD4/CCR5 bond probability distributions produced by viral particles with randomly distributed gp120. Example bond probability distributions show a combination of Phase II and Phase III-like distributions with bimodal probability nodes, which correspond to the gp120 distribution on the viral surface. The viral particles contained 15 gp120 trimers.
(0.84 MB EPS)

Text S1 A detailed, step-by-step description of how viral adhesion was modeled.
(0.08 MB DOC)
