# Supplementary material for: Organization of Cellular Receptors into a Nanoscale Junction during HIV-1 Adhesion
Source: PLoS Comput Biol. 2010 Jul 15;6(7):e1000855. doi: 10.1371/journal.pcbi.1000855 (PMC2904768; doi:10.1371/journal.pcbi.1000855)
Supplement: Text S1 — A detailed, step-by-step description of how viral adhesion was modeled. (0.08 MB DOC) [file pcbi.1000855.s002.doc]

**Supplemental Material for**

**Organization of cellular receptors into a nanoscale junction during HIV-1 adhesion**

Terrence M. Dobrowsky^1^, Brian R. Daniels^1^, Robert F. Siliciano^2^, Sean X. Sun^1,3,4^, & Denis Wirtz^1,4*^

^1^Department of Chemical and Biomolecular Engineering, The Johns Hopkins University, 3400 North Charles Street, Baltimore, Maryland 21218, USA

^2^Howard Hughes Medical Institute and Department of Medicine, The Johns Hopkins School of Medicine, 733 North Broadway Street, Baltimore, Maryland 21205, USA

^3^Department of Mechanical Engineering, The Johns Hopkins University, 3400 North Charles Street, Baltimore, Maryland21218, USA

^4^ Johns Hopkins Physical Sciences in Oncology Center, The Johns Hopkins University, 3400 North Charles Street, Baltimore, Maryland21218, USA

The combined system of virion, plasma membrane, and viral and cellular receptors studied here, is modelled as a succession of discrete states. The transition to new states is assumed to be a Markov process and computed using the local-steady state approximation to the Fokker-Planck equation. This approximation calculates the rate of transition between the current state and subsequent adjacent state using the total change in energy that accompanies the progression from one discrete state to another. Specifically, a favourable change in energy (e.g. the relaxation of a bond between viral proteins and cellular receptors) results in an increased transition rate and an unfavourable change in energy (e.g. the deformation of the plasma membrane) results in a decreased transition rate from one state to the next.

The following is a step-by-step description of how the interaction between a viral particle and a flexible plasma membrane was performed. Briefly, all variables are declared and a see for the pseudo random number generator is obtained using the computer clock. All variable input by the user are obtained at this time through external .txt files.

- All source code was written in Fortran.
- Declare all variable type and array dimension.
- Call a seed for the pseudo random number generator (PRNG) which is based off the computer clock time. Starting each program at a different time gives a different string of random numbers for each simulation. All random numbers are uniformly distributed between 0 and 1 unless otherwise indicated.
- Read all input files, which determine values for
  - Number of iterative state changes for system to run during initialization
  - Number of iterative state changes for system to run over simulation
  - Number of discrete plasma membrane points, P, resulting in a PM PxP dimension
  - XY distance between each discrete PM point
  - Change in vertical position (Δz-step) used to calculate forward rate for movement of each PM point
  - Diffusion constant used to calculate forward rate for movement of each PM point
  - PM coefficient of elasticity used to calculate the Energy associated with its deformation
  - PM surface tension used to calculate the Energy associated with its deformation
  - Radius of viral particle
  - Change in XYZ position (Δxyz-step) used to calculate forward rate for movement of the viral particle in space
  - Diffusion constant used to calculate forward rate for movement of the viral particle
  - Degree of angular change associated with rotation of the viral particle
  - Density of CD4 and CCR5 on the plasma membrane
  - Diffusion constants used to calculate forward rate for movement of each CD4 and CCR5 protein
  - Stable CD4 bond characteristics including, unstressed k_off_, x*, ΔG*, κ_m_, according to the model described by Szabo and Hummer as well as the CD4 protein length – These values are for the initial CD4 bond micromechanics observed with CD4 expression alone in Dobrowsky et al, JVI, 2008.
  - Unstable CD4 bond characteristics including, unstressed k_off_, x*, ΔG*, κ_m_, according to the model described by Szabo and Hummer as well as the CD4 protein length – These values are for the most unstable CD4 bond micromechanics observed with CD4 and CCR5 expression in Dobrowsky et al, JVI, 2008.
  - CCR5 bond characteristics including, unstressed k_off_, x*, ΔG*, κ_m_, according to the model described by Szabo and Hummer as well as the CCR5 protein length
  - gp120 protein characteristics including, when allowed, the diffusion constant used to calculate forward rate for movement of the gp120 spikes on the viral surface, the number of gp120 trimers on the viral surface, the angle from viral surface normal allowed for bond formation, the length of the gp120 protein, and whether or not gp120 will be allowed to diffuse (given here as either 0 = fixed, or diffuse = 1).
- Create all output files where data will be written to during the simulation, including physical locations of virion, gp120 trimers, CD4 and CCR5 proteins, and z-position of each discrete plasma membrane points. Also saved are the bond number, lengths and energies. System properties were saved every 100,000 system state changes.

**Initializing virion system parameters**

- Establish empty array for all possible bonds between gp120-CD4 and gp120-CCR5 as well as formed bonds between gp120-CD4 and gp120-CCR5. These will be of lengths (NumberOfgp120*SystemDimension*SystemDimension) as well as (NumberOfgp120*3), respectively. This way, the index of the possible bond arrays and formed bond arrays can be used to easily refer to specific possible bonds or existing bonds.
- Establish arrays governing virion and viral protein positions. Virion is modeled as a hard sphere with radius, Vr. An array of evenly distributed gp120 positions about the virion were calculated according to,

************************************************************************

DO k = 1,gpdim

h = -1 + 2*(real(k)-1)/(real(gpdim)-1)

phi(k,1) = ACOS(h)

IF (k .EQ. 1) THEN

theta(k,1) = 0

END IF

IF (k .EQ. gpdim) THEN

theta(k,1) = 0

END IF

IF (k .NE. 1 .AND. k .NE. gpdim) THEN

N = theta(k-1,1) + 3.6/SQRT(gpdim*(1-h**2))

theta(k,1) = MOD(N,(2*3.1415))

END IF

END DO

- - - Here, gpdim is the number of gp120 trimers

************************************************************************

- - For systems with randomly distributed gp120 surface units about the surface, theta was chosen randomly with uniform distribution between 0 and 2π while phi was chosen randomly between 0 and π according to the probability density distribution P(phi) = 0.5*sin(phi). The gp120 trimers were not allowed to be placed with overlapping volumes. If one was randomly placed in an overlapping region with another, the new trimer was discarded and another was randomly placed until physical restrictions were satisfied.

************************************************************************

CALL random_number(ran1)

CALL random_number(ran2)

CALL random_number(ran3)

theran = 2*3.1415*ran1

phiran = 3.1415*ran2

Pran = 0.5*sin(3.1415/2)*ran3

IF (Pran <= 0.5*sin(phiran)) THEN

X = vr*cos(theran)*sin(phiran)

Y = vr*sin(theran)*sin(phiran)

Z = vr*cos(phiran)

END IF

************************************************************************

**Initialize plasma membrane (PM) system parameters**

- - Establish an array that will serve as the plasma membrane (PM) which will be SystemDimensionXSystemDimension in size with discrete positions of the PM evaluated at deltaPM where SystemDimension and deltaPM are user inputs. Initially, all heights (z-position) of discrete points are 0. Here for example, SystemDimension is 40 and deltaPM is 5 nm making a 200x200 nm plasma membrane.
  - Calculate the initial probability of every discrete point on the PM to move up or down the z-step size (input by user). Forward rates for positive movements and negative movements are stored in separate arrays. Two discrete positions along the edge of the system have movement rates equal to 0 for ease in calculating first and second derivatives in the membrane curvature during the simulation. Total rate constants are calculated for upward and downward movement by summing all individual rates of discrete positions. For ease of use, the current energies are saved in an array for use later. Energies for discrete points on the PM were calculated throughout the simulation using,

************************************************************************

hx = hpm(x+1,y) - hpm(x-1,y)

hx = hx / (2*delpm)

hy = hpm(x,y+1) - hpm(x,y-1)

hy = hy / (2*delpm)

hxx = hpm(x+1,y)-2*hpm(x,y)+hpm(x-1,y)

hxx = hxx / delpm**2

hyy = hpm(x,y+1)-2*hpm(x,y)+ hpm(x,y-1)

hyy = hyy / delpm**2

hxy = (hpm(x+1,y+1) -

+ hpm(x-1,y+1) - hpm(x+1,y-1) +hpm(x-1,y-1))/ (2*delpm)**2

g = 1 + hx**2 + hy**2

E(x,y) = kappa / (2 * g**(5/2))*

+ (hxx *(1+hy**2) + hyy*(1+hx**2)-

+ 2*hxy*hx*hy)**2 + gamma*SQRT(g)

- - - Here hx is the first derivative of the local membrane height with respect to x and hxx is the second derivative. The same is for hy and hyy. Also, hxy is the second order derivative with respect to x and y. Kappa is the membrane elasticity coefficient, gamma is the surface tension and E is the local energy of the discrete PM point at x,y.
    - To calculate the change in energy associated with the movement of a discrete point on the PM at (x,y), the change in energy for discrete locations from (x-2):(x+2) and (y-2):(y+2) were summed and used in the solution to the Fokker-Planck equation described in the text. For an initially flat plasma membrane at z = 0 everywhere, all discrete points will have an equal probability of movement.

************************************************************************

- - For a preset number of system state changes, allow the system to change with only the movement of the plasma membrane with no interaction with the viral particle in order to establish a random surface of interaction.
    - Add the values of PM movement upward and downward directions (k_up_ and k_down_) to get a total system forward rate (K).
    - It is important to maintain the order in which these properties were summed. Here, all individual forward rates for discrete positions along a single y position of the PM were added (stepping from 0 in the positive x direction) until all discrete positions were accounted for. This was first done with the positive movement array, and then with the negative movement array.
    - Obtain an evenly distributed random value between 0 and K (R).
    - If the value R is less than k_up_ then there will be a positive movement, if it is more than k_up_ then there will be a negative movement.
    - Once the entity which will change is determined (PM up or down) calculate a new R specific for that state, as in, if R is more than k_up_, subtract k_up_ from R to obtain sR (a random position along the coordinate 0 to k_down_, specific for that state).
    - Iteratively subtract (according to the same order in which they were added) the rates of movement for the individual discrete positions until the discrete position corresponding to sR is established. Knowing the location of PM movement and its direction, the next state is determined.
  - Apply the movement of that PM position.
  - Update the energy array at the discrete PM point moved as well as those discrete points surrounding the newly altered PM point, as their energies will also be affected by its movement.
  - Update the individual PM point diffusion rates for the moved membrane position and all those around it affected by its change in z-position. Do this for upward and downward diffusion.
  - If the discrete PM point moved had a protein located there or in any of its nearest neighbors, the diffusion of that protein to any of the nearest neighbor positions is updated.

**Initialize receptor system parameters**

- - Establish an array of protein positions on the PM by randomly distributing CD4 and CCR5. Positions on the PM were determined by evenly distributed PRNG between 0 and the membrane length dimension. Proteins were not allowed to be placed with overlapping volumes, if an overlapping protein was randomly chosen, it was discarded and a new randomly chosen position was used until physical restrictions were satisfied.
  - Build arrays governing the diffusion rates for all CD4 and CCR5 proteins. Given the Cartesian coordinates used here, four arrays were built for CD4 and CCR5, governing the +/- x and +/- y directional movement.
    - Diffusion constants are determined by calculating the 3D distance from one discrete PM point to its four nearest neighbors.
    - If another protein exists in the position that a current protein would diffuse to, that diffusion is set to zero.
  - For conditions in which the experimentally determined instability is imposed a binary instability array of size SystemDimensionXSystemDimension is established. If the initial randomly distributed protein positions result in instability, i.e. if a CD4 has a CCR5 located at any of its nearest neighbor positions, the location of CD4 is marked in the instability array as ‘1’ and will dictate that the program use the ‘unstable’ bond micromechanics (input by user) to calculate possible bond forward rate constants and bond dissociation rates accordingly.

**Initialize remaining system parameters**

- Calculate forward rate constants for all bonds, gp120-CD4, gp120-CD4(unstable) and gp120-CCR5. According to,
- After the initialization period. Establish the virion position above the PM. To expedite the initial adhesion, the bottom of the virion is located a distance above the PM equal to the length of CD4 and gp120.
- Calculate the initial probability of the virion moving upward or downward directions a z-step distance, input by user.
  - For ease in calculation, XY movement of the virion is not considered until bonds have formed between it and the plasma membrane.
- Calculate the probability of virion rotation a curvilinear distance, input by user.
- Check for initial possible CD4 bond formations.
  - Calculate the distance from each CD4 to each gp120. If within acceptable bond formation distances, record the XY position of CD4, the specific gp120 capable of binding to it, the distance between them, the energy associated for that possible bond and the forward rate for that bond in the array designated for possible gp120-CD4 bonds.
    - Acceptable bond positions are determined by comparing the distance between points on the virion and plasma membrane anchoring the proteins (d_total_) to upper and lower limits. The upper limit is the length of gp120, CD4 and twice the distance of the energy minimum to rupture as recorded experimentally (due to the parabolic assumption for energy landscape) while the lower limit for acceptable bond distance is the length of gp120 and CD4. In addition, bonds were not allowed to form with gp120 trimers beyond a imposed angle from normal of the viral surface, input by user.
  - Sum all possible forward rates and obtain a rate constant for the state when a CD4 bond is formed (K_CD4_).
- Recall a random seed for the PRNG as before.
- If diffusion of gp120 is allowed, calculate the diffusion of each individual gp120 trimer positions a preset curvilinear distance. The proteins can move independently as opposed to in concert as with total virion rotation. If gp120 trimer positions come within a preset distance of one another, the forward rate of movement is set equal to 0.

**Allow system to progress through states while virion and membrane interact**

- For a preset number of iterations, calculate system changes for all new states allowed including the movement of PM points upward and downward, the movement of the virion upward and downward, the movement of CD4 or CCR5, form a CD4 bond, break a CD4 bond, form a CCR5 bond, break a CCR5 bond, spin the virion largely if the no bonds are formed, rotate the virion slightly if bonds are formed and finally move gp120 trimer points if diffusion is allowed.
  - The future states here are chosen in the same manner as earlier. Total forward rates are summed (K) and a uniformly distributed random value between 0 and K is chosen, R. The order with which forward rates are summed is important. For instance, the probability that any discrete plasma membrane point will move positively up is added to the probability that any point will move negatively down, is added to the probability that the virion will move, etc.
  - kPlasma_Membrane_up + k Plasma_Membrane_down + k Virion_Move+ k CD4_Bond_Make+ kCD4_Bond_Break+ etc. = R
  - R is used to choose which entity within the system will be changed and calculate the random state variable (sR) to determine what individual unity of an entity type will be changed (i.e. first it will be decided that a CD4 protein will diffuse then the individual CD4 to move will be chosen as well as the direction).
    - If R is greater than kPlasma_Membrane_up + k Plasma_Membrane_down but is less than kPlasma_Membrane_up + k Plasma_Membrane_down + k Virion_Move then sR = R - kPlasma_Membrane_up - k Plasma_Membrane_down and in the same way that R was used to determine which entity will change, sR is used to determine which direction the virion will move.
- If the future state chosen is a movement of the PM in an upward or downward direction.
  - Use sR and the individual values for discrete PM points to determine which point will move to a new position. Move that PM position. If that point is involved in a bond, update that bonds length, energy and dissociation rate.
  - Update the energy of the discrete PM point moved.
  - Update the individual PM point forward rates for the moved membrane position and all those around it affected by its change in z-position. Do this for upward and downward movement. If there is a protein located at the discrete PM point moved, the change in energy that would occur should the PM point be moved again is used to calculate the forward rate of that movement. Check to see if the positive movement of the PM discrete position moves the PM into space occupied by the virion, if so, set the positive direction forward rate equal to 0.
  - If the discrete PM point moved had a protein located there or in any of its nearest neighbors, the forward rates for diffusion of that protein to any of its nearest neighbors is updated. If there is a bond formed on that protein the diffusion is scaled according to the change in energy between the current state and the possible one.
  - If an unbound protein is located at the altered PM point, check to see if it can now form a possible bond with a gp120 trimer as well is if the new position disallows the possibility of a bond to be formed. If previously possible bonds are no longer allowed, delete relevant information from the possible bond array. If new possible bonds are discovered or if preexisting ones are still allowed, update the possible bond array with the new the distance between proteins, the energy associated for that possible bond and the forward rate for that bond.
  - If that point is involved in a bond, calculate how the new bond micromechanics affect the forward rate of virion rotation and movement in all directions.
  - If diffusion of gp120 is allowed, and that point moved is involved in a bond, how the diffusion rate of that gp120 is affected and recalculate.
- If the future state chosen is the movement of the virion.
  - Change the array governing the 3D location of the hard sphere representing the virion, the location of all gp120 z-locations and the center of the virion accordingly.
  - Change the length, energy and dissociation rate of every bond that is exists.
  - Change the forward rate for diffusion of all proteins involved in a bond.
  - Calculate the new diffusion rates for the virion to move in all directions. If the move downward forces the virion to move through or equal to the PM height, set the rate of the virion todiffuse negatively equal to zero.
  - Clear all possible CD4 and CCR5 bonds. Check for all possible CD4 and CCR5 bonds for each gp120.
  - If diffusion of gp120 is allowed, and a gp120 trimer is involved in a bond, recalculate the diffusion of that gp120 based on the new bond micromechanics.
  - Calculate the new upward and downward diffusion of discrete points of the PM involved in bonds according to the new bond micromechanics.
- If the future state chosen is the movement of CD4 or CCR5.
  - sR is used to determine which specific protein will move in which specific direction. Also, if there are too many free proteins and one is on the periphery of the system that protein diffuses out of the system and is not replaced. Otherwise a protein is moved. Determine that proteins nearest neighbors, if CD4 is adjacent to CCR5, update the array controlling instability.
  - If that protein is involved in a bond, recalculate the bonds length, energy and dissociation rate. Recalculate the PM discrete point forward rates for movement of the protein according to the new bond micromechanics. Also, recalculate the forward rate for the rotation and translocation of the virion according to the new micromechanics.
  - Check neighbors of the new protein location. If the new protein location restricts movement previously allowed for other proteins due to physical restrictions, set the forward rate of movement for those proteins to zero for the appropriate direction.
  - If that protein is not involved in a bond, check if that new position allows for any possible bond formations and clear all possible bond formations for that protein at its last position.
  - If diffusion of gp120 is allowed, and the protein moved is involved in a bond, recalculate the diffusion of gp120 according to the new bond micromechanics.
- If the future state chosen is the formation of a CD4 bond.
  - Use sR to determine which possible CD4 bond will be formed and update the bond array accordingly. Delete all possible bonds to be formed involving that CD4 protein.
  - Update the virion diffusion forward rates according to the new bond micromechanics, as well as the rotation of the virion.
  - Update the upward and downward PM point diffusion of that involved with the newly formed bond according to the bond micromechanics.
  - Update the forward rates of diffusion for the protein involved with the newly formed bond according to the bond micromechanics.
  - Create a new CD4 protein on the periphery of the system by randomly choosing a point for it to enter. Using a uniformly distributed PRN, place the CD4 protein only the edge of the system such that its volume does not overlap with another protein. Update the diffusion forward rates for this protein.
  - If this is the first bond formed, set the forward rate of the more dramatic spinning of the virion equal to zero.
  - If diffusion of gp120 is allowed, recalculate the diffusion rate of the gp120 involved with the new bond according to the new bond micromechanics.
- If the state chosen is the destruction of a CD4 bond.
  - Use sR to determine which CD4 bond will be deleted and remove it from the bond array.
  - Update the forward rates of diffusion for the virion according to the new bond micromechanics.
  - Update the forward rates of diffusion for the CD4 protein that was involved with the deleted bond.
  - Update the upward and downward PM point diffusion of that involved with the newly formed bond according to the bond micromechanics.
  - Check to see if the newly available CD4 can possibly form a bond and update the possible bond array accordingly.
  - Check to see if the newly available gp120 can possibly form a new bond and update the possible bond array accordingly.
  - Delete the possible CCR5 bonds that the presence of the recently deleted CD4 bond allowed.
  - If diffusion of gp120 is allowed, update the diffusion of the newly free gp120 according to new bond micromechanics.
- If the future state chosen is the formation of a CCR5 bond.
  - Use sR to determine which possible CCR5 bond will be formed and update the bond array accordingly. Delete all possible bonds to be formed involving that CCR5 protein.
  - Update the upward and downward virion diffusion according to the new bond micromechanics.
  - Update the upward and downward PM point diffusion of that involved with the newly formed bond according to the bond micromechanics.
  - Update the diffusion of the protein involved with the newly formed bond according to the bond micromechanics.
  - Update the diffusion rate of the virion according to the new bond micromechanics.
  - Create a new CCR5 protein on the periphery of the system by randomly choosing a point for it to enter. If no free points currently exist, wait for the state of CCR5 movement when there is a free point.
  - Set the diffusion rate of the more dramatic spinning of the virion to zero.
  - If diffusion of gp120 is allowed, recalculate the diffusion rate of the gp120 involved with the new bond.
- If the state chosen is the destruction of a CCR5 bond.
  - Use sR to determine which CCR5 bond will be deleted and remove it from the bond array.
  - Update the diffusion rate of the virion according to the new bond micromechanics.
  - Update the diffusion of the CCR5 protein that was involved with the deleted bond.
  - Update the upward and downward PM point diffusion of that involved with the newly formed bond according to the bond micromechanics.
  - Check to see if the newly available CCR5 can possibly form a bond and update the possible bond array accordingly.
  - Check to see if the newly available gp120 can possibly form a new bond and update the possible bond array accordingly.
  - If diffusion of gp120 is allowed, update the diffusion of the newly free gp120 according to new bond micromechanics.
- If the state chosen is the movement of the virion gp120 positions.
  - Use sR to determine which direction the virion will rotate and update all bonds currently formed according to their new length, energy and dissociation rates.
  - Recalculate the diffusion rate for virion rotation according to the new bond micromechanics.
  - Check all gp120 trimer units for possible bond formations.
  - If diffusion of gp120 is allowed, recalculate all diffusion rates according to new gp120 positions.
  - Update the diffusion rates for movement of all proteins involved in currently formed bonds according to the new bond micromechanics.
  - Update the upward and downward diffusion rates for each discrete PM points currently involved in bonds according to their new micromechanics.
- If diffusion of gp120 is allowed and that is the state chosen.
  - Use sR to determine which individual gp120 unit is going to be moved and in which direction. Move that gp120 unit.
  - Update any bonds involved with that gp120 unit.
  - Calculate the diffusion rate of all gp120 units.
  - If the gp120 unit that was moved was involved with a bond, recalculate the upward and downward diffusion rates of the virion, the upward and downward diffusion rates of discrete points of the PM involved with bonds.
  - Delete possible bonds to be formed with the moved gp120 unit and check for any new possible bonds to be formed. Update the possible bond array accordingly.
  - Calculate the diffusion rate for virion rotation according to the new bond micromechanics if the moved gp120 unit is involved in a bond.

****Note: For all processes below, before calculating energies or lengths for bonds, if unstable CD4 bond properties are to be used, check the array governing whether the bond in question will use stable or unstable bond properties, then proceed accordingly.**

**Calculating forward rates for possible bond formation.**

- Calculate the distance between the discrete point of the PM where the protein is located and the point on the virion surface where the gp120 trimer is located (d).
- Subtract the length of the PM protein in question and the length of gp120 from d (d_bond).
- If d_bond is between 0 and 2(x*), where x* is specific to the type of bond in question, then the bond is possible and k_forward_ is applied.

**Calculating forward rates for bond dissociation.**

- Calculate the distance between the discrete point of the PM where the protein is located and the point on the virion surface where the gp120 trimer is located (d).
- Subtract the length of the PM protein in question and the length of gp120 from d (x).
- Use x to calculate the free energy according to,
- Use Eb to calculate the forward rate of bond dissociation according to,

**Calculating forward rates for rotation of the virion.**

- Calculate the new positions of gp120 trimers on the virion given a hypothetical rotation by a degree input by the user
- Use the new positions of gp120 trimers to calculate the new distance between each trimer position on the viral surface and the protein location on the PM (d).
- Subtract the length of gp120 and the protein in question (*x*).
- Use *x* to calculate the new free energy of the bond according to,
- Sum the *E_b_* for all bonds affected by virion rotation and in conjunction with the free energies of bonds prior to the hypothetical rotation calculate the forward rate for viral rotation according to
- If no bonds exist, Δ*E* is zero

**Calculating forward rates for translocation of the virion.**

- Calculate the new positions of gp120 trimers on the virion given a hypothetical translocation of the virion, Δ*l*, input by user.
- Use the new positions of gp120 trimers to calculate the new distance between each trimer position on the viral surface and the protein location on the PM (d).
- Subtract the length of gp120 and the protein in question (*x*).
- Use *x* to calculate the new free energy of the bond according to,
- Sum the *E_b_* for all bonds affected by translocation and in conjunction with the free energies of bonds prior to the hypothetical translocation calculate the forward rate for viral rotation according to
- If no bonds exist, Δ*E* is zero

**Calculating forward rates for cellular protein diffusion.**

- Check if a second protein is occupying the space that another protein will move to.
  - If there is no physical restriction, proceed
  - If another protein is occupying that space, set the forward rate for that protein diffusion in that direction equal to 0.
- Calculate the distance between the current protein position and the future one.
- If that protein is involved in a bond, calculate the future energy of the bond using the future protein location.
  - Calculate the new distance between the trimer position on the virion and the new protein location on the PM (d).
  - Subtract the length of gp120 and the protein in question (*x*).
  - Use *x* to calculate the new free energy of the bond according to,
  - Calculate Δ*E* using the current energy of the bond and then the forward rate of protein diffusion using
  - If no bonds exist, Δ*E* is zero

**Calculating forward rates for viral protein diffusion.**

- Check if a second protein is occupying the space that another protein will move to.
  - If there is no physical restriction, proceed
  - If another protein is occupying that space, set the forward rate for that protein diffusion in that direction equal to 0.
- Use the predefined distance, input by user.
- If that protein is involved in a bond, calculate the future energy of the bond using the future protein location.
  - Calculate the new distance between the new trimer position on the virion and the protein location on the PM (d).
  - Subtract the length of gp120 and the protein in question (*x*).
  - Use *x* to calculate the new free energy of the bond according to,
  - Calculate Δ*E* using the current energy of the bond and then the forward rate of protein diffusion using
  - If no bonds exist, Δ*E* is zero
